# GIFT4-augmented B cells for melanoma immunotherapy

**DOI:** 10.1186/2051-1426-2-S3-P9

**Published:** 2014-11-06

**Authors:** Jiusheng Deng, Andrea Pennati, Shala Yuan, Ragini Kudchadkar, David Lawson, Jacques Galipeau

**Affiliations:** 1 Winship Cancer Institute, Emory University, Atlanta, Georgia, USA

## 

Melanoma is the most dangerous type of skin cancer and one of the fastest growing cancers in the USA. Immunotherapy, by enhancing patient's immune system, has become an attractive approach for melanoma treatment due to the rapid drug resistance to chemotherapy. B cells are a heterogeneous cell population in the host immune system functionally as antibody-producers, antigen-presenting cells or regulatory cells. In the tumor microenvironment, B cells are among the heavy tumor-infiltrating immune cells. But B cell's role in anti-tumor immunity remains controversial. Our lab has recently developed a novel GM-CSF and IL-4 fusion cytokine named GIFT4 (GMCSF and IL4 Fusion Transgene). This technology builds upon the successful development of "fusokine" platform [GIFT15] (*Nature Medicine*, 2009, 15: 1038-45). We discovered GIFT4 possess novel gain-of-function of immune activities, elicits robust B cell response and consequent killer T cell immunity against melanoma. GIFT4 protein has potent immune signaling activities, induces hyper-phosphorylation of STAT1, 3 and 5 in B cells, and reprograms B cells from melanoma patients into CD40^+^CD80^+ ^CD83^+^CD86^+ ^antigen-presenting cells profiled by flow cytometry. Luminex assay reveals that GIFT4-B cells secrete substantial amount of IL-2, innate cytokines IL-6 and GM-CSF, chemokines CCL2, CCL3, CCL4 and CCL5, and adhesion molecule ICAM-1, but not IL-10 and IFN- γ. Those GIFT4-B cells robustly promote sustained *ex vivo *expansion of tumor-killing T cells that are IFN-γ^+^, NKG2D^+ ^Granzyme B^+ ^and granulysin^+^, and produce Fas ligand and TRAIL. Moreover, human GIFT4-B cell licensed cytotoxic T cells kill human melanoma cells in *in vitro *and in NSG immune deficient mice. We expect that GIFT4, as an anti-melanoma agent, will provide a novel strategy and opens a new avenue for human B cell-based immunotherapy against melanoma. We propose that GIFT4-B cells from melanoma patients could serve as a potential immunotherapeutic agent for personalized melanoma cell immunotherapy.

